# A novel homozygous mutation in the *DNAAF3* gene leads to severe asthenozoospermia and teratospermia

**DOI:** 10.1111/jcmm.70092

**Published:** 2024-09-17

**Authors:** Dongjia Chen, Guoqing Fan, Yan Xu, Peng Luo, Qinyun Chen, Xuren Chen, Zexin Guo, Xianqing Zhu, Yong Gao

**Affiliations:** ^1^ Reproductive Medicine Center, Guangdong Provincial Key Laboratory of Reproductive Medicine, Guangdong Provincial Clinical Research Center for obstetrical and gynecological diseases, The First Affiliated Hospital Sun Yat‐sen University Guangzhou China

**Keywords:** asthenozoospermia, *DNAAF3*, male infertility, murine model, PCD, teratospermia

## Abstract

Primary ciliary dyskinesia (PCD) is an autosomal recessive genetic disorder characterized by ultrastructural defects in the cilia or flagella of cells, causing respiratory abnormalities, sinusitis, visceral transposition, and male infertility. DNAAF3 plays an important role in the assembly and transportation of axonemal dynein complexes in cilia or flagella and has been shown to be associated with PCD. To date, only two cases of PCD with infertility associated with *DNAAF3* mutations have been reported, and no mouse models for this gene have been successfully constructed. This study was conducted on an infertile Chinese male patient with a history of bronchitis. Examination of the patient's semen revealed severe asthenozoospermia and teratospermia. Whole exome sequencing revealed a new homozygous loss‐of‐function *DNAAF3* mutation. CRISPR‐Cas9 gene‐editing technology was used to construct the same mutation in C57/B6 mice, revealing that homozygous C57/B6 mice were characterized by severe hydrocephalus and early death. The results of this study expand the mutation spectrum of *DNAAF3* and confirm its correlation with PCD pathogenesis. This study provides new insights on the mechanisms underlying male infertility related to *DNAAF3* mutation and PCD.

## 1 INTRODUCTION

Infertility is a major issue worldwide and the rate of incidence is increasing annually. Currently, infertility affects at least 7% of men worldwide, and male‐related factors are estimated to contribute to 30%–50% of couples' infertility.^1^, ^2^ Asthenozoospermia is an important cause of male infertility. According to the fifth edition of the World Health Organization's (WHO) Laboratory Manual for the Examination and Processing of Human Semen, asthenozoospermia refers to the progressive motility rate of sperm being lower than the reference value (32%).^3^ The movement of sperm depends on the regular swinging of the sperm flagella, which is powered by the central axoneme running through the body of the flagella. Cross‐sections of the axoneme reveal a ‘9 + 2’ structure, consisting of nine doublet microtubules surrounding the central pair of single microtubules.^4^ Outer (ODA) and inner (IDA) dynein arms diverge from nine pairs of peripheral doublet microtubules and are responsible for converting the chemical energy of adenosine triphosphate (ATP) into mechanical force, which generates power for the sperm flagella.^5^ Genetic mutations involved in flagellar assembly and motility regulation are recognized as important causes of asthenozoospermia.^6^, ^7^, ^8^ In addition, factors affecting sperm maturation or ejaculation processes may also impact sperm motility, such as reproductive tract infections,^9^ varicocele,^10^ lifestyle^11^ and toxic substances,^12^ among others.

Primary ciliary dyskinesia (PCD) is an autosomal recessive genetic disease that affects approximately 1:10000 people worldwide.^13^, ^14^ It is characterized by ciliary dyskinesia and results in a variety of clinical manifestations, including chronic bronchitis, bronchiectasis, interstitial pneumonia, sinusitis, secretory otitis media, situs inversus and so on. Sperm flagella are a special type of cilia and an abnormal sperm flagellar structure can cause the sperm to lose motility. Therefore, PCD is often associated with male infertility.^15^ Although the ciliary pathogenesis of the PCD respiratory phenotype has been widely studied, there are relatively few reports on the impact of PCD on male infertility.^16^ According to a systematic review of the validated monogenic causes of human male infertility by the International Male Infertility Genomics Consortium (IMIGC) in 2020, eight PCD‐related genes are at least moderately linked to male infertility, namely, *CCDC39*, *CCDC40*, *DNAAF2*, *DNAAF4*, *DNAAF6*, *LRRC6*, *RSPH3* and *SPEF2*.^17^

The *DNAAF3* gene is located on human chromosome 19q13 and contains 12 exons. It encodes axonemal assembly factor 3, a protein required for the assembly of axonemal dyneins and the assembly and trafficking of the axonemal dynein complex.^18^, ^19^ Protein expression abnormalities caused by *DNAAF3* gene mutations have been shown to lead to assembly defects in the axonemal dynein complex, further causing abnormal ciliary structure and function, which can lead to respiratory system abnormalities, sinusitis, visceral transposition, etc.^18^, ^19^ The dynein arm is a molecular motor for sperm flagellar movement. When *DNAAF3* gene mutations cause structural abnormalities, the outer and inner dynein arms may be absent at the ultrastructural level, resulting in immotile sperm or severe asthenozoospermia, thus affecting male reproductive potential. However, there are very few reports on PCD in infertile males with *DNAAF3* mutations and on their semen characteristics, with only two cases reported thus far.^19^, ^20^ Furthermore, no disease model has been established for this gene in mice.

In this study, we identified a novel *DNAAF3* mutation in a Chinese male suffering with PCD and severe asthenozoospermia. Using CRISPR‐Cas9 gene‐editing technology, we developed a mouse model with homozygous mutations that mimicked the identified variation. This study expands the spectrum of *DNAAF3* mutations and supplements the pool of male infertility cases attributed to *DNAAF3* mutations. Moreover, using mouse models, this study confirmed its correlation with PCD pathology. These findings offer valuable insights into the mechanisms underlying male infertility associated with *DNAAF3* and PCD.

## 2 METHODS

### 2.1 Patients

**FIGURE 1:**
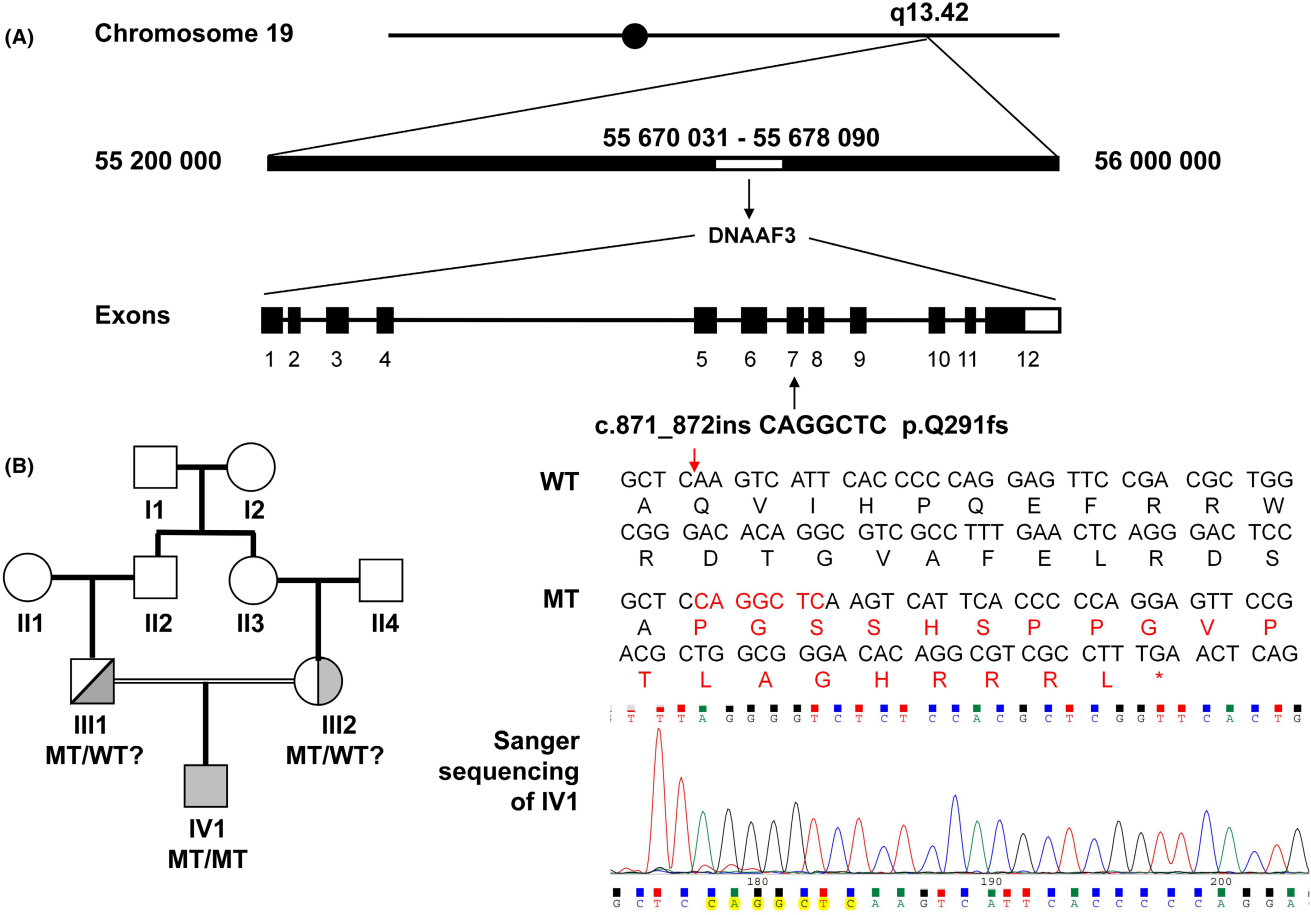
An illustration of the mutation site in the *DNAAF3* gene observed in the patient in this study (A), as well as a pedigree chart (B). (A) Whole exome sequencing (WES) revealed that the patient (**IV1**) had a homozygous insertion of seven nucleotides (c.871_872ins CAGGCTC, p.Q291fs) in exon 7 of the *DNAAF3* gene on chromosome 19. This represents a novel homozygous mutation, which results in the premature termination of translation after the insertion site, leading to a missense of 20 amino acids. Sanger sequencing confirmed this homozygous mutation. (B) The parents of the patient (**III1** and **III2**) are first cousins, indicating a possible heterozygous *DNAAF3* mutation; however, Sanger sequencing was not performed to validate this assumption.

A 24‐year‐old male patient with primary infertility who was treated at the Reproductive Medicine Center of the First Affiliated Hospital of Sun Yat‐sen University was enrolled in this study (Figure 1). The patient's parents were first cousins (consanguineous marriage). The patient also had a history of bronchitis. There was no clear acquired aetiology, such as orchitis, epididymitis, cryptorchidism, severe varicocele, history of exposure to reproductive toxic drugs, including chemotherapy or radiation or history of exposure to chemical toxins or radiation that could cause reproductive damage. This study was conducted in accordance with the principles of the Declaration of Helsinki and was approved by the Independent Ethics Committee for Clinical Research and Animal Trials of the First Affiliated Hospital of Sun Yat‐sen University (approval number: [2021]326; date of approval: May 12th, 2021). Written informed consent was obtained from the patient.

### 2.2 Semen examination

The patient, following standard clinical semen collection requirements, abstained from ejaculation for 2–7 days and provided a semen sample through masturbation. According to the laboratory manual on Human Semen Analysis and Processing by the World Health Organization (WHO, 5th edition), the obtained semen sample was analysed using computer‐assisted sperm analysis (CASA) to determine sperm quantity and vitality parameters. Subsequently, semen smears and staining were performed to evaluate sperm morphology. Additionally, sperm vitality was measured using the hypotonic swelling (HOS) test (normal range: ≥50%^3^, ^21^). Tests for genital tract inflammation were conducted, including semen leukocyte peroxidase (POX) staining (normal range: ≤1 × 10^6^/mL,^3^, ^21^) and seminal plasma elastase detection (normal range: <290 ng/mL^22^).

### 2.3 Whole exome sequencing

Peripheral blood samples were used for the extraction, fragmentation, and library construction of genomic DNA (gDNA) using the QIAamp DNA Blood Mini Kit (QIAGEN), Covaris S220 focused ultrasonicator (Covaris) and NEBNext Ultra II DNA Library Prep Kit for Illumina (NEB), respectively. A TruSeq Exome Enrichment Kit (Illumina) was used to enrich the coding regions and intron/exon boundaries. Sequencing was performed on the NextSeq 550DX (Illumina) platform using the Truseq SBS Kit V4‐HS reagent kit (Illumina). The sequence reads were aligned to the reference genome (GRCh37/hg19) using the Burrows‐Wheeler Aligner. Single‐nucleotide variants and small insertions/deletions (InDels) were identified using SAM tools (http://samtools.sourceforge.net/). Filtering, annotation and pathogenicity analyses of mutations were performed using conventional analytical methods, as described previously.^20^

### 2.4 Sanger Sequencing

After designing primers corresponding to the mutation site of *DNAAF3* (NM_001256714), the target sequence was amplified by PCR using genomic DNA from the patient as a template. PCR products were Sanger sequenced using conventional methods.^23^

### 2.5 Mouse model construction

**FIGURE 2:**
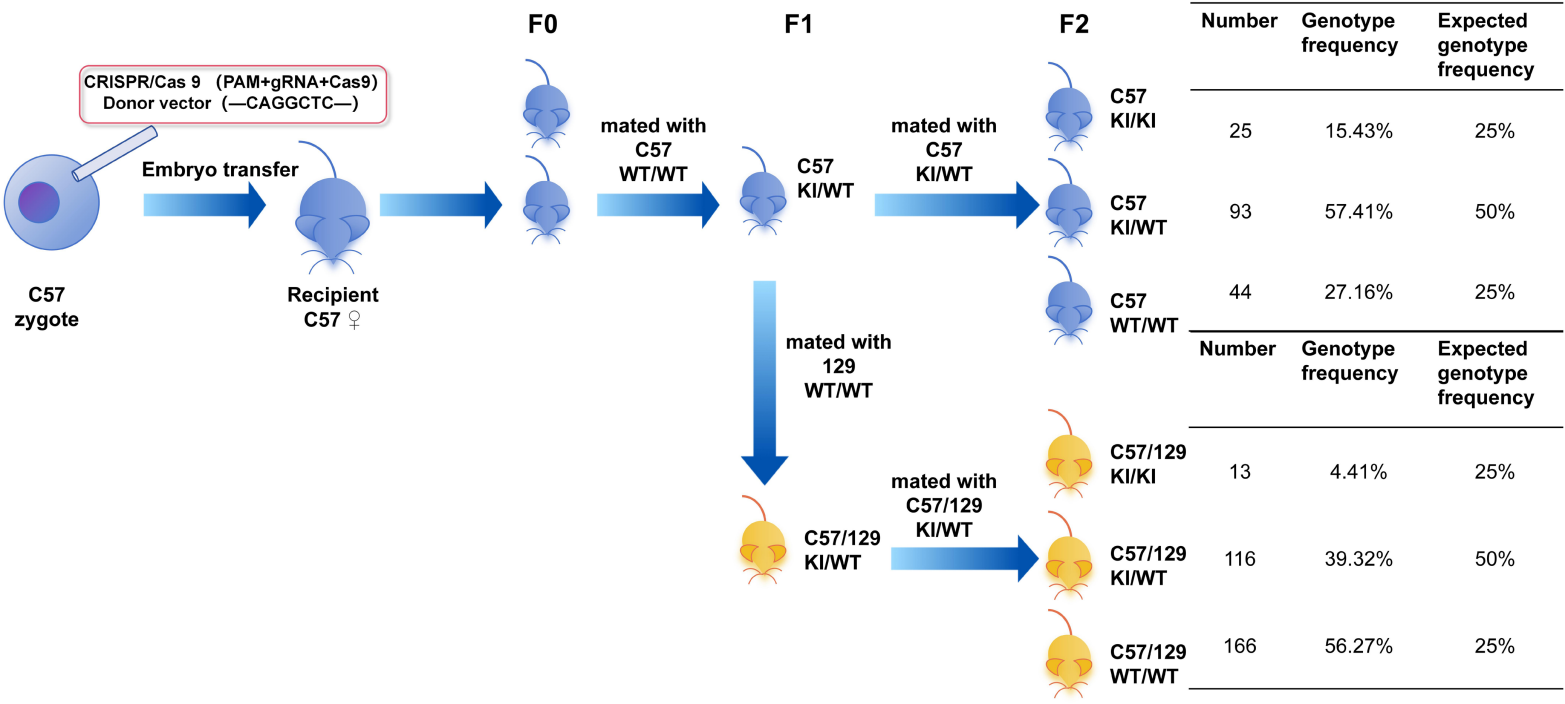
Workflow for the construction of *DNAAF3* homozygous mutant mice. CRISPR/Cas9 technology was used to modify C57 zygotes, introducing a 7‐nucleotide insertion (c.871_872ins CAGGCTC, p.Q291fs) into the *DNAAF3* gene to create the same mutation as that observed in the patient. After zygote manipulation, the embryos were transferred, resulting in heterozygous F1 offspring. Heterozygous F1 mice were then bred to obtain homozygous F2 offspring. In the F2 generation, the genotypic distribution of wild‐type (WT) mice, heterozygotes, and homozygotes deviated from the Mendelian inheritance ratios with percentages of 15, 57% and 27%, respectively. To obtain hybrid mice with both C57 and 129 genetic backgrounds, heterozygous F1 mice were crossed with WT 129 strains. The F1 generation mice obtained from this cross possessed both C57 and 129 genetic backgrounds and were heterozygous for the targeted mutation. Further mating among these F1 mice resulted in homozygous F2 offspring. In the F2 generation, the genotypic distribution of WT mice, heterozygotes, and homozygotes deviated from the Mendelian inheritance ratios with percentages of 4.41%, 39.32% and 56.27%, respectively.

The mouse model construction process is illustrated in Figure 2. The mice were kept in a facility meeting specific pathogen free standards, with a 12‐hour light/dark cycle. In vitro‐transcribed sgRNA (Table 1) and donor vectors were constructed and microinjected into fertilized C57BL/6JGpt mouse zygotes with Cas9, sgRNA and a donor for homologous recombination. Seven‐base insertions (c.871_872ins CAGGCTC, p.Q291fs) in *DNAAF3* were generated to produce the same mutation as that in the patient. After injection, the surviving zygotes were transferred to pseudopregnant female mice, and the mice were allowed to give birth. The F0 progeny born from the recipient mice were tail‐ and toe‐clipped at 5–7 days old, genotyped by PCR and sequenced using the primers shown in Table 2 to confirm their genotype. After reaching sexual maturity, F0 heterozygous mice were crossed with wild‐type mice. F1 progeny were genotyped by PCR and sequenced to confirm their genotype. Heterozygous F1 mice were crossed to obtain homozygous C57 mice in the F2 generation.

**TABLE 1:** gRNA sequence information.

gRNA name	gRNA sequence (5′‐3′)	PAM
S1	ATTCCTGGATATGGATAACT	TGG
S2	ATAACTTGGGCCTGGAGTAG	AGG

**TABLE 2:** PCR primers for *DNAAF3* mutant mice.

Primer name	Sequence	Product size
F1	ATGAAGCTGCATGATCGAGGG	WT:599 bp targeted:606 bp
R1	CACCTTGACTGGTTGTCCGTTTC	

After crossing heterozygous C57 mice with homozygous 129 wild‐type mice, the F1 progeny were used to extract genomic DNA for PCR and sequenced to confirm their genotype. Heterozygous F1 mice carrying both C57 and 129 genetic backgrounds were crossed to obtain homozygous mice in the F2 generation that possessed both C57 and 129 genetic backgrounds simultaneously.

Genotyping was performed at 5–7 days after birth, and no deaths were observed at that time. The lung and brain tissues of mice were stained with haematoxylin and eosin (H&E). Additionally, the testicular tissues were stained with both H&E and periodic acid Schiff (PAS) stain.

All procedures were approved by the Independent Ethics Committee for Clinical Research and Animal Trials of the First Affiliated Hospital of Sun Yat‐sen University and performed in accordance with the People's Republic of China regulations concerning the use of animals in research.

### 2.6 Statistical analysis

Statistical analyses were performed using SPSS software (version 22.0; IBM Corp., Chicago, IL, USA). Student's *t*‐test was used to compare body weight and testicular volume between wild‐type and mutant mice. The Pearson chi‐squared test was used to determine any statistical differences between actual and expected genotype frequencies in the F2 generation. *p* < 0.05 was considered statistically significant.

## 3 RESULTS

### 3.1 Semen examination

Table 3 provides a detailed display of the patients' semen examination results. Semen volume, pH, total sperm count and sperm concentration were normal. However, severe asthenozoospermia was observed, with a sperm progressive motility (PR) of 0% and non‐progressive motility (NP) of 0.8%. Additionally, approximately 99.1% of the sperm exhibited abnormal morphology; the total head abnormality rate was 96.5%, neck abnormality rate was 30.1%, and tail abnormality rate was 64.2%, with 52.4% of the sperm being short tailed. Based on this, we calculated the patient's multiple anomalies index (MAI) as 1.92. This value was higher than the mean MAI of fertile men (1.58 ± 0.2).^3^, ^21^ However, both the fifth and sixth editions of the WHO laboratory manual do not specify a range of reference values for MAI, and the sixth edition of the WHO laboratory manual states that no sharp limit exists between fully fertile and subfertile men.^3^, ^21^ The sperm HOS test revealed a vitality rate of 45%, suggesting a decrease in sperm viability in the patient. The patient's semen POX staining was 0.15 × 10^6^/mL and seminal plasma elastase was 42.9 ng/mL, which were both within normal ranges, indicating no reproductive tract infection in the patient.

**TABLE 3:** Detailed results of the patient's semen examination.

Semen parameters		Reference Values of the WHO Laboratory Manual for the Examination and Processing of Human Semen	
		5th edition	6th edition
Semen volume (mL)	3.0	1.5^a^	1.4^a^
Semen PH	7.5	≥7.2	≥7.2
Sperm concentration (×10^6^/mL)	78.0	15^a^	16^a^
Total sperm count (×10^6^)	234.0	39^a^	39^a^
Progressive motility (%)	0.0	32^a^	30^a^
Non‐progressive motility (%)	0.8	1^a^	1^a^
Total motility (%)	0.8	40^a^	42^a^
Immotility (%)	99.2	20^a^	20^a^
Sperm morphology			
Normal (%)	0.9	4^a^	4^a^
Abnormal (%)	99.1		
Total abnormal head (%)	96.5		
Total abnormal tail (%)	64.2		
Total abnormal neck (%)	30.1		
Multiple anomalies index (MAI)	1.92^b^		
Sperm tail morphology			
Absent (%)	5.2		
Short (%)	52.4		
Coiled (%)	0.0		
Curling (%)	6.6		
Irregular width (%)	0.0		
Normal (%)	35.8		
Morphometric parameters of sperm			
Length of head (μm), mean ± S.E.M.	3.9 ± 0.9		
Width of head (μm), mean ± S.E.M.	2.4 ± 0.4		
Width of neck (μm), mean ± S.E.M.	1.0 ± 0.6		
Acrosomal region of sperm head: total surface of sperm head (×100), mean ± S.E.M.	38.5 ± 12.8		
Vitality (%)	45	58	54
Genital tract inflammation			
Peroxidase‐positive leukocytes (10^6^/mL)		<1	<1
Seminal plasma elastase detection			

^a^ 5th centile.

^b^ This value is higher than the mean MAI of fertile men (1.58 ± 0.2).^3^, ^21^ However, both the fifth and sixth editions of the WHO laboratory manual do not specify a range of reference values for MAI, and the sixth edition of the WHO laboratory manual states that no sharp limit exists between fully fertile and subfertile men.^3^, ^21^

### 3.2 WES and Sanger sequencing

Whole‐exome sequencing (WES) revealed a novel mutation site in exon 7 of *DNAAF3*, characterized by a homozygous insertion of seven bases (c.871_872ins CAGGCTC, p.Q291fs). This mutation was identified as a frameshift mutation. This variant was not found in the normal control population of the Exome Aggregation Consortium (ExAC) (http://exac.broadinstitute.org/) database or the 1000 Genomes Project (http://www.1000genomes.org/data) database. Sanger sequencing was performed to confirm this mutation in the patient; however, no familial verification was performed.

### 3.3 Pathogenicity analysis of *DNAAF3* mutation

*DNAAF3* (c.871_872ins CAGGCTC, p.Q291fs) was identified as a loss‐of‐function (LOF) variant. This mutation resulted in the incorrect translation of 20 amino acids, followed by premature termination (Figure 1). According to American College of Medical Genetics and Genomics (ACMG) guidelines,^24^ this variant has been classified as pathogenic. Computational analysis using the online variant pathogenicity prediction tool MutationTaster (http://www.mutationtaster.org/) indicated that this mutation is ‘disease causing’, with a probability of 100%.

### 3.4 *DNAAF3* mutant mice

**FIGURE 3:**
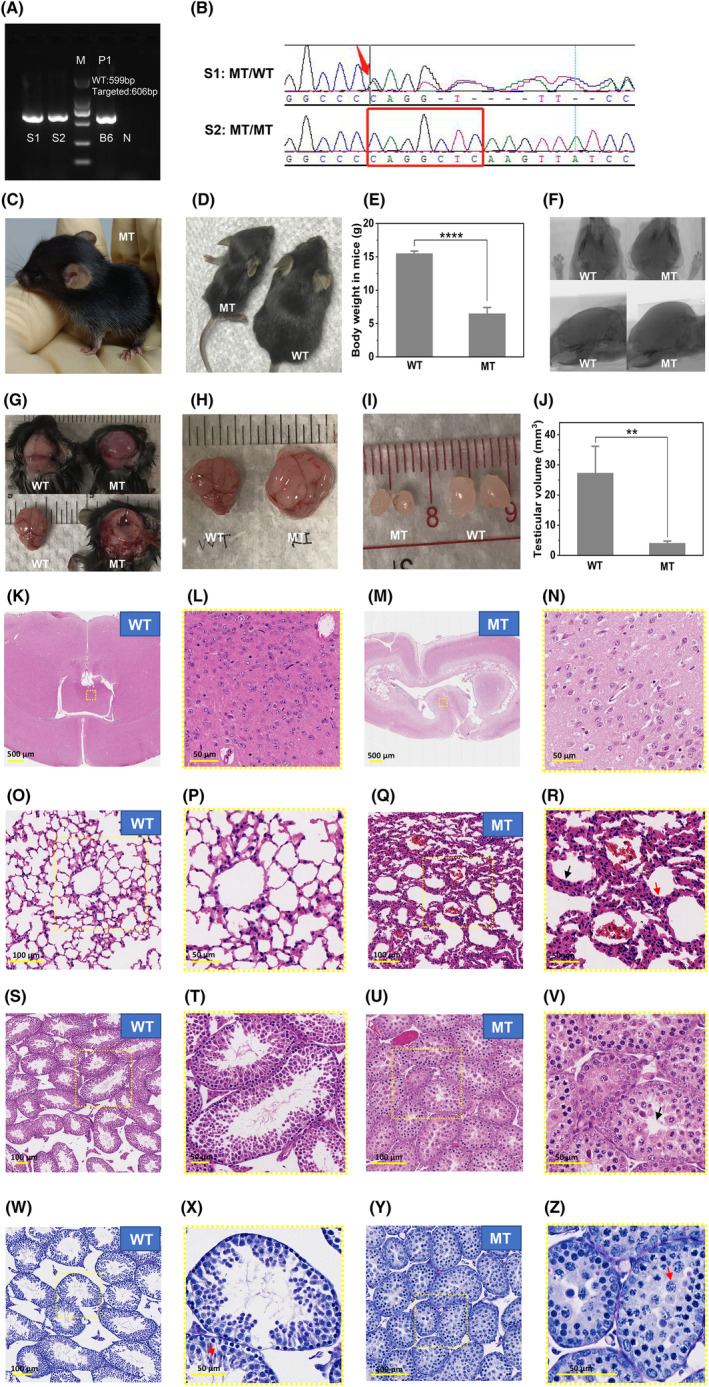
Phenotypic characteristics of *DNAAF3* homozygous mutant mice. (A) Electrophoresis results of mutant mice. Sample S1 is a heterozygote, sample S2 is a homozygote, sample B6 is wild‐type control genomic DNA (gDNA), and sample N is the blank control. (B) Sanger sequencing results of mutant mice. Mutant mice carry a 7‐base insertion mutation (c.871_872ins CAGGCTC, p.Q291fs) in the *DNAAF3* gene, which is identical to the mutation observed in the patient. (C–E) Phenotypic features of homozygous and wild‐type mice. Compared to wild‐type mice, homozygous mice exhibit growth defects, with enlarged heads and smaller bodies. The body weight of homozygous mice was significantly lower than that of wild‐type mice (6.49 ± 0.93 vs. 15.50 ± 0.37 g, *p* = 0.0015) (The original data can be found in Table S1). (F) Micro computed topography (MicroCT) images of the skulls of homozygous and wild‐type mice. The skull outline is enlarged in homozygous mice. (G‐H) Results of anatomical examination of the skulls of homozygous and wild‐type mice. Compared to wild‐type mice, homozygous mice exhibit increased brain volume, enlarged ventricles, and thinning of the cerebral cortex. (I, J) Results of anatomical examination of the testes of homozygous and wild‐type mice. The testes size is significantly reduced in homozygous mice compared to that in wild‐type mice (8.90 ± 0.74 vs. 27.28 ± 4.03 mm^3^, *p* < 0.0001) (The original data can be found in Table S2). (K–N) Normal morphology of brain tissue in homozygous and wild‐type mice stained with haematoxylin & eosin (scale: 500 and 50 μm). (O–R) Haematoxylin and eosin (H&E) staining of lung tissue sections (scale: 100 and 50 μm). Compared to wild‐type mice, homozygous mice exhibit increased proliferation and a dense arrangement of alveolar epithelial cells, congested and dilated blood vessels (black arrow in R), and thickening of alveolar septa (red arrow in R). (S–V) H&E staining of testicular tissue sections (scale: 100 and 50 μm). Both homozygous and wild‐type mice exhibit prepubertal characteristics without mature sperm. In homozygous mice, the diameter of the seminiferous tubules was reduced, the walls were thin, and the lumens were narrow or occluded with a decrease in the hierarchy and quantity of spermatogenic cells. (W–Z) Periodic acid Schiff (PAS) staining of testicular tissue sections (scale: 100 and 50 μm). The germ cell types in wild‐type mice were more diverse than those in homozygous mice. Elongated spermatids were observed in the lumen line (red arrow in X), indicating the movement of the nucleus from a central position in the cell so that the acrosome approaches the surface of the cytoplasm and points toward the basal membrane of the tubule, signifying Stage 8 spermatids. Whereas, in homozygous mice, most of the germ cells were pachytene spermatocytes (red arrow in Z) and acrosomic granules were not observed, which indicated that the stage of germ cells was before the round spermatid stage.

Using CRISPR/Cas9, modifications were made to the C57 zygotes. After embryo transfer, heterozygous F1 offspring were obtained and crossed to obtain homozygous F2 offspring. The genotype frequencies of wild‐type mice, heterozygotes, and homozygotes in the F2 generation were 15.43%, 57.41% and 27.16%, respectively, which exhibited no significant difference with expected frequencies (25%, 50% and 25%) (*p* = 0.09). Figure 3 displays the phenotypic characteristics of the mutant mice. When compared to wild‐type mice, homozygous mice exhibited enlarged heads and weak bodies (Figure 3C,D). The body weight of homozygous mice was significantly lower than that of wild‐type mice (6.49 ± 0.93 vs. 15.50 ± 0.37 g, *p* = 0.0015) (Figure 3E) (The original data can be found in Table S1). Micro‐computed tomography revealed an expanded cranial contour (Figure 3F). The anatomical results revealed that the brains of homozygous mice were larger (Figure 3G,H), ventricles were more dilated (Figure 3G), cerebral cortex was thinner (Figure 3G), and testicular volume was significantly lower than that of wild‐type mice (8.90 ± 0.74 vs. 27.28 ± 4.03 mm^3^, *p* < 0.0001) (Figure 3I,J) (The original data can be found in Table S2). H&E staining of brain tissue from homozygous mice revealed normal morphology (Figure 3K–N). The H&E ‐stained lung tissue sections of homozygous mice exhibited increased proliferation and dense arrangement of alveolar epithelial cells, congested and dilated blood vessels, and thickened alveolar septa compared to those in wild‐type mice (Figure 3O–R).

The testicular tissue sections stained with H&E (Figure 3S–V) showed prepubertal characteristics in both wild‐type and homozygous mice, with no mature sperm. To determine the exact stage of germ cells with the development of the acrosome cap or the morphology of the younger generation of spermatids, the testicular tissues were subjected to PAS staining. As shown in Figure 3W–Z, the germ cell types in wild‐type mice were more diverse than those in homozygous mice. Elongated spermatids were observed in the lumen line (red arrow in Figure 3X), indicating the movement of the nucleus from a central position in the cell so that the acrosome approaches the surface of the cytoplasm and points toward the basal membrane of the tubule, signifying stage 8 spermatids. Whereas in homozygous mice, most of the germ cells were pachytene spermatocytes (red arrow in Figure 3Z), and acrosomic granules were not observed, which indicated that the stage of germ cells was before the round spermatid stage. The diameter of the seminiferous tubules in homozygous mouse testes decreased, the walls became thinner, and the lumens narrowed or became blocked, with a decrease in the number and hierarchy of germ cells (Figure 3V,Z).

All of the homozygous mice died before postnatal day 21 (PND21).

By crossing the C57 heterozygous and wild‐type 129 strains, heterozygous mice with both C57 and 129 genetic backgrounds were obtained in the F1 generation and were subsequently mated to obtain homozygous F2 offspring. The genotype frequencies of wild‐type mice, heterozygotes, and homozygotes in the F2 offspring of the C57/C57 cross were 4.41%, 39.32% and 56.27%, respectively, which were significantly different from the expected frequencies (*p* < 0.001). Homozygous mice with both C57 and 129 genetic backgrounds had a high mortality rate, and all died before PND21.

## 4 DISCUSSION

The axonemal dynein complex, comprising the ODA and IDA, is responsible for generating and regulating the beating of cilia and flagella.^25^ The axonemal dynein arm is first assembled in the cytoplasm and then delivered to the axoneme during ciliogenesis. Members of the dynein axonemal assembly factor (Dnaaf) protein family are involved in the pre‐assembly and stability of dynein arms before they are transported to the cilium; mutations in *DNAAF* family genes can cause partial or complete loss of the dynein arm, resulting in structural and functional defects of cilia, and is closely related to the pathogenesis of PCD.^19^, ^26^, ^27^, ^28^ Three *DNAAF* family members (*DNAAF2*, *DNAAF4* and *DNAAF6*) play significant roles in male fertility.^17^ *DNAAF3* has been identified as one of the PCD‐related pathogenic genes, and based on the Global Variome shared LOVD database, as of 7th July 2023, a total of 212 common variants have been reported (https://databases.lovd.nl/shared/genes/DNAAF3). In this study, we identified a novel mutation in the *DNAAF3* gene through WES of samples from an infertile Chinese male; bioinformatics analysis showed that this variant was highly pathogenic, and may have led to PCD‐related phenotypes, such as bronchitis and severe asthenozoospermia, in this male. This study also presented the semen examination results of the patient and established a homozygous mutant mouse model. The results of this study expand the spectrum of *DNAAF3* mutations and provide important clues for investigating the mechanisms underlying *DNAAF3*‐related male infertility associated with PCD.

The first reported case of *DNAAF3* mutation dates back to 2012.^19^ Mitchison et al. reported the clinical features of 10 patients carrying *DNAAF3* mutations, with each patient displaying typical symptoms of PCD, including chronic cough, recurrent chest infections, sinusitis, severe lung disease, bronchiectasis, and hearing loss.^19^ Four of the 10 patients also presented with situs inversus. Additionally, the study reported the first case of infertility associated with a *DNAAF3* mutation, in which the patient was diagnosed with immotile sperm; however, more detailed semen analysis results were not provided. More recently, Wan et al. reported a second case of *DNAAF3* mutation‐related male infertility, with the patient presenting with sinusitis and recurrent lower respiratory tract infections, but no situs inversus.^20^ The semen examination indicated severe asthenozoospermia (1.7% progressive motility and 0.49% non‐progressive motility) but normal morphology. Transmission electron microscopy and immunostaining analysis of the patient's sperm revealed the complete absence of the ODA and partial absence of the IDA. The patient produced offspring through intracytoplasmic sperm injection (ICSI).

The newly identified case of homozygous *DNAAF3* mutation‐related infertility in our study also presented with a history of bronchitis and infertility but without situs inversus. Semen examination of the patient indicated a normal sperm count, but approximately 99.2% of the sperm were immotile and approximately 99.1% exhibited morphological abnormalities, suggesting severe asthenozoospermia and teratospermia. This finding is similar to the semen examination results reported for mutations in the *DNAAF1/2/3/4/6/7* family proteins, where the sperm count is usually within the normal range, but with impaired motility and varying degrees of morphological abnormalities.^20^, ^25^, ^29^, ^30^ Although Wan et al. reported normal sperm morphology in their *DNAAF3* mutation case,^20^ we observed that reduced flagellar length was an important characteristic of sperm morphology in the patient with *DNAAF3* mutation in our study. Previously reported human patients with *DNAAF2*, *4*, *6* and *7* mutations also exhibited significantly decreased flagellar length, and transmission electron microscopic examination of the sperm flagella revealed defects in or the absence of the ODA and IDA.^25^, ^29^, ^30^ *Chlamydomonas*, an algal genus widely used to study flagellar motility, with DNAAF3 mutations also displayed shortened flagella.^19^ Evidence has suggested that shortened flagella in single‐cell algal species occurs due to the loss of various types of axonemal dyneins.^31^, ^32^, ^33^ Although our study did not analyse the ultrastructure of the patient's sperm, Wan et al.'s research validated the presence of ODA and IDA abnormalities in the sperm flagella of *DNAAF3* mutation patients using electron microscopy.^20^ These findings suggest that the presence of the ODA and IDA is crucial for the stability of sperm flagella.

Animal models can provide direct evidence supporting an association between mutant genes and specific diseases. Previous studies have attempted to generate animal models with *DNAAF3* mutations. Mitchison et al. found that flagella in *DNAAF3* mutant *Chlamydomonas* were shortened, lacked motility and exhibited defects in dynein assembly. Knocking down *dnaaf3* in zebrafish disrupted dynein arm assembly and ciliary movement, resulting in phenotypes associated with PCD, such as kidney cysts, hydrocephalus, disrupted otolith development and lateral defects.^19^ In addition, research conducted by Zur lage et al. using the *Drosophila* model revealed that mutations in the *DNAAF3* homologue gene CG17669 can lead to hearing loss in larvae and coordination issues in adult flies. Moreover, the study found that male *Drosophila* flies with these mutations were unable to reproduce, as their chordotonal neuron cilia and sperm flagella lacked dynamic protein arms and exhibited axoneme damage.^18^ These studies have provided strong evidence confirming the correlation between *DNAAF3* gene mutations and PCD. However, no researchers have successfully developed a *DNAAF3* mutant mammalian model, such as a mouse model.

In the present study, we used CRISPR‐Cas9 technology to generate C57BL/6 mice carrying the same homozygous *DNAAF3* mutation. The proportion of homozygotes in the F2 offspring was significantly lower than the Mendelian inheritance ratio, which may be related to the intrauterine loss of homozygotes during pregnancy, which is similar to the results obtained after constructing *DNAAF5* mutant mice.^34^ This indicates that *DNAAF3* is essential for embryonic growth and development and that its functional impairment is embryonically lethal. Furthermore, the homozygous mutant mice exhibited severe hydrocephalus and early lethality. These results are consistent with the descriptions of *DNAAF1*, *DNAAF2*, *DNAAF4*, and *DNAAF5* mutant mice, all of which exhibited severe hydrocephalus shortly after birth and died prior to PND21.^27^, ^34^, ^35^, ^36^ Motile cilia in the ependymal layer play a crucial role in facilitating the flow of cerebrospinal fluid, and their dysfunction can lead to the accumulation of cerebrospinal fluid and congenital hydrocephalus.^37^, ^38^ In humans, PCD‐associated hydrocephalus is less common than the typical symptoms, such as recurrent respiratory infections, male infertility, and situs inversus. The *DNAAF3* mutant male patient included in this study only exhibited bronchitis and infertility phenotypes without evidence of hydrocephalus. However, hydrocephalus is frequently observed in PCD mouse models, possibly due to the significant anatomical and physiological differences in brain development between humans and mice.^39^

Different strains of mice exhibit varying susceptibilities to and severities of PCD‐associated hydrocephalus. C57BL/6 and FVB/N mice are highly sensitive to PCD‐related hydrocephalus and are prone to early death, while 129S6/SvEv and C3H/He mice appear to have lower sensitivity and may reach normal lifespans.^39^ Therefore, in this study, heterozygous C57BL/6 mice were crossed with 129 background mice to obtain *DNAAF3* mutant mice exhibiting both genetic backgrounds. However, none of these mice survived until sexual maturity in the present study. The preliminary results of the mouse model constructed in this study provide evidence for a correlation between *DNAAF3* and the phenotype of PCD‐associated hydrocephalus.

Noteworthily, PAS staining of the testicular tissue revealed stage 8 germ cells in wild‐type mice, whereas in homozygous mice, the seminiferous tubules exhibited a reduced number and stratification of spermatocytes and their development was arrested before the round spermatid stage. This finding suggests that the gene mutation may cause a blockade in germ cell development in mice; however, further evidence is needed to confirm this. We cannot exclude the possibility that the spermatogenic blockade observed in homozygous mice might be a secondary effect resulting from multi‐system pathologies such as hydrocephalus and pulmonary oedema or a primary alteration induced by the gene mutation itself. Moreover, we observed that the lumens of seminiferous tubules in homozygous mice were narrowed or obstructed. However, owing to a lack of well‐preserved efferent duct tissue samples, we were unable to perform haematoxylin and eosin staining combined with immunofluorescence to investigate potential efferent duct multicilial defects, as described by Hoque et al.^40^ Future research is warranted to further explore whether the structural abnormalities are caused by increased pressure or other factors associated with efferent duct multicilial defects.

This study has several limitations. First, owing to the lack of patient cooperation, we were unable to obtain blood samples from the parents for pedigree verification. Thus, we could not determine whether the mutation was inherited from the parents. Additionally, owing to the patient's noncompliance, we could not obtain an adequate number of semen samples; therefore, we did not perform scanning and transmission electron microscopy on the patient's sperm. The effect of this mutation on the ultrastructure of human sperm, thus, requires further investigation. Second, we were unable to conduct functional gene validation studies using testicular tissue and sperm samples from the patient. These functional validation experiments could help determine the localisation, quantification, and extent of the impact of the mutation on DNAAF3 and expression of other related proteins, providing more direct evidence to support the pathogenicity of the mutation and its association with infertility. Third, the infertile male subject in this study did not undergo assisted reproductive treatments; therefore, we could not observe whether ICSI treatment could help the patient produce healthy offspring. Fourth, regarding the construction of the *DNAAF3* mutant mouse model, we did not conduct ultrastructural examinations of mouse neurons and respiratory cilia. In addition, we were unable to successfully obtain *DNAAF3* mutant mice that survived until adulthood. Therefore, the role of the gene in sperm tail development could not be investigated. Fifth, we did not explore the underlying reasons for the defects in testicular structure in *DNAAF3* mutant mice in the present study.

## 5 CONCLUSIONS

In summary, research on the association between *DNAAF3‐* and PCD‐related infertility remains limited among the reported *DNAAF* family gene mutations. In this study, we identified a new *DNAAF3* mutation through the genetic analysis of an infertile male in China and attempted to generate a corresponding mouse model, which suggests the relevance of *DNAAF3* in the pathogenesis of PCD. Despite the limitations, the findings of this study provide new insights into the relationship between *DNAAF3* mutations and male infertility associated with PCD. Future studies should aim to improve the functional validation and mouse models to uncover the mechanisms underlying *DNAAF3* gene mutations in male infertility and may play a role in the development of better diagnostic and therapeutic strategies as well as the provision of genetic counselling support for patients with PCD‐related infertility.

## AUTHOR CONTRIBUTIONS

**Dongjia Chen:** Data curation (equal); formal analysis (equal); visualization (equal); writing – original draft (equal). **Guoqing Fan:** Formal analysis (equal); methodology (equal); project administration (equal); writing – original draft (equal). **Yan Xu:** Conceptualization (equal); methodology (equal); project administration (equal); supervision (equal); writing – original draft (equal). **Peng Luo:** Investigation (equal); project administration (equal); software (equal); validation (equal). **Qinyun Chen:** Data curation (equal); formal analysis (equal); software (equal); validation (equal). **Xuren Chen:** Data curation (equal); investigation (equal); validation (equal). **Zexin Guo:** Methodology (equal); project administration (equal). **Xianqing Zhu:** Data curation (equal); methodology (equal). **Yong Gao:** Conceptualization (equal); data curation (equal); funding acquisition (equal); resources (equal); supervision (equal); validation (equal); writing – review and editing (equal).

## CONFLICT OF INTEREST STATEMENT

The authors have no conflict of interest to declare.

## CONSENT

Written informed consent was obtained from the patient.

## Supporting information


Table S1.

## Data Availability

Data supporting the findings are available from the corresponding author upon reasonable request.

## References

[1] Eisenberg ML , Esteves SC , Lamb DJ , et al. Male infertility. Nat Rev Dis Primers. 2023;9:49. 37709866 10.1038/s41572-023-00459-w

[2] Krausz C , Riera‐Escamilla A . Genetics of male infertility. Nat Rev Urol. 2018;15:369‐384. 29622783 10.1038/s41585-018-0003-3

[3] World Health Organization . WHO Laboratory Manual for the Examination and Processing of Human Semen. 5th ed. World Health Organization, Switzerland; 2010.

[4] Inaba K . Molecular architecture of the sperm flagella: molecules for motility and signaling. Zool Sci. 2003;20:1043‐1056. 10.2108/zsj.20.1043 14578564

[5] Lehti MS , Sironen A . Formation and function of sperm tail structures in association with sperm motility defects. Biol Reprod. 2017;97:522‐536. 29024992 10.1093/biolre/iox096

[6] Tu C , Wang W , Hu T , Lu G , Lin G , Tan YQ . Genetic underpinnings of asthenozoospermia. Best Pract Res Clin Endocrinol Metab. 2020;34:101472. 33191078 10.1016/j.beem.2020.101472

[7] Oud MS , Houston BJ , Volozonoka L , et al. Exome sequencing reveals variants in known and novel candidate genes for severe sperm motility disorders. Hum Reprod. 2021;36:2597‐2611. 34089056 10.1093/humrep/deab099 PMC8373475

[8] Tang S , Wang X , Li W , et al. Biallelic mutations in CFAP43 and CFAP44 cause male infertility with multiple morphological abnormalities of the sperm flagella. Am J Hum Genet. 2017;100:854‐864. 28552195 10.1016/j.ajhg.2017.04.012 PMC5473723

[9] Shahrokhi SZ , Salehi P , Alyasin A , Taghiyar S , Deemeh MR . Asthenozoospermia: cellular and molecular contributing factors and treatment strategies. Andrologia. 2020;52:e13463. 31680293 10.1111/and.13463

[10] Baazeem A , Belzile E , Ciampi A , et al. Varicocele and male factor infertility treatment: a new meta‐analysis and review of the role of varicocele repair. Eur Urol. 2011;60:796‐808. 21733620 10.1016/j.eururo.2011.06.018

[11] Verón GL , Tissera AD , Bello R , et al. Impact of age, clinical conditions, and lifestyle on routine semen parameters and sperm kinematics. Fertil Steril. 2018;110:68‐75.e64. 29980266 10.1016/j.fertnstert.2018.03.016

[12] Lafuente R , García‐Blàquez N , Jacquemin B , Checa MA . Outdoor air pollution and sperm quality. Fertil Steril. 2016;106:880‐896. 27565259 10.1016/j.fertnstert.2016.08.022

[13] Rubbo B , Lucas JS . Clinical care for primary ciliary dyskinesia: current challenges and future directions. Eur Respir Rev. 2017;26:170023. 28877972 10.1183/16000617.0023-2017 PMC9489029

[14] Lucas JS , Burgess A , Mitchison HM , et al. Diagnosis and management of primary ciliary dyskinesia. Arch Dis Child. 2014;99:850‐856. 24771309 10.1136/archdischild-2013-304831 PMC4145427

[15] Goutaki M , Meier AB , Halbeisen FS , et al. Clinical manifestations in primary ciliary dyskinesia: systematic review and meta‐analysis. Eur Respir J. 2016;48:1081‐1095. 27492829 10.1183/13993003.00736-2016

[16] Sironen A , Shoemark A , Patel M , Loebinger MR , Mitchison HM . Sperm defects in primary ciliary dyskinesia and related causes of male infertility. Cell Mol Life Sci. 2020;77:2029‐2048. 31781811 10.1007/s00018-019-03389-7 PMC7256033

[17] Houston BJ , Riera‐Escamilla A , Wyrwoll MJ , et al. A systematic review of the validated monogenic causes of human male infertility: 2020 update and a discussion of emerging gene‐disease relationships. Hum Reprod Update. 2021;28:15‐29. 34498060 10.1093/humupd/dmab030 PMC8730311

[18] Zur Lage P , Xi Z , Lennon J , et al. The *drosophila* orthologue of the primary ciliary dyskinesia‐associated gene, DNAAF3, is required for axonemal dynein assembly. Biol Open. 2021;10:10. 10.1242/bio.058812 PMC8565470 34553759

[19] Mitchison HM , Schmidts M , Loges NT , et al. Mutations in axonemal dynein assembly factor DNAAF3 cause primary ciliary dyskinesia. Nat Genet. 2012;44(381–389):s381‐s382. 10.1038/ng.1106 PMC3315610 22387996

[20] Wan F , Yu L , Qu X , et al. A novel mutation in PCD‐associated gene DNAAF3 causes male infertility due to asthenozoospermia. J Cell Mol Med. 2023;27:3107‐3116. 37537752 10.1111/jcmm.17881 PMC10568663

[21] World Health Organization . WHO Laboratory Manual for the Examination and Processing of Human Semen. 6th ed. World Health Organization; 2021.

[22] Zorn B , Virant‐Klun I , Meden‐Vrtovec H . Semen granulocyte elastase: its relevance for the diagnosis and prognosis of silent genital tract inflammation. Hum Reprod. 2000;15:1978‐1984. 10966999 10.1093/humrep/15.9.1978

[23] Crossley BM , Bai J , Glaser A , et al. Guidelines for Sanger sequencing and molecular assay monitoring. J Vet Diagn Invest. 2020;32:767‐775. 32070230 10.1177/1040638720905833 PMC7649556

[24] Richards S , Aziz N , Bale S , et al. Standards and guidelines for the interpretation of sequence variants: a joint consensus recommendation of the American College of Medical Genetics and Genomics and the Association for Molecular Pathology. Genet Med. 2015;17:405‐424. 25741868 10.1038/gim.2015.30 PMC4544753

[25] Aprea I , Raidt J , Höben IM , et al. Defects in the cytoplasmic assembly of axonemal dynein arms cause morphological abnormalities and dysmotility in sperm cells leading to male infertility. PLoS Genet. 2021;17:e1009306. 33635866 10.1371/journal.pgen.1009306 PMC7909641

[26] Omran H , Kobayashi D , Olbrich H , et al. Ktu/PF13 is required for cytoplasmic pre‐assembly of axonemal dyneins. Nature. 2008;456:611‐616. 19052621 10.1038/nature07471 PMC3279746

[27] Tarkar A , Loges NT , Slagle CE , et al. DYX1C1 is required for axonemal dynein assembly and ciliary motility. Nat Genet. 2013;45:995‐1003. 23872636 10.1038/ng.2707 PMC4000444

[28] Raidt J , Wallmeier J , Hjeij R , et al. Ciliary beat pattern and frequency in genetic variants of primary ciliary dyskinesia. Eur Respir J. 2014;44:1579‐1588. 25186273 10.1183/09031936.00052014

[29] Sun M , Zhang Y , Yang J , et al. Novel compound heterozygous DNAAF2 mutations cause primary ciliary dyskinesia in a Han Chinese family. J Assist Reprod Genet. 2020;37:2159‐2170. 32638265 10.1007/s10815-020-01859-7 PMC7492306

[30] Zhou L , Li Z , Du C , et al. Novel dynein axonemal assembly factor 1 mutations identified using whole‐exome sequencing in patients with primary ciliary dyskinesia. Mol Med Rep. 2020;22:4707‐4715. 33174003 10.3892/mmr.2020.11562 PMC7646867

[31] Lin H , Zhang Z , Guo S , et al. A NIMA‐related kinase suppresses the flagellar instability associated with the loss of multiple axonemal structures. PLoS Genet. 2015;11:e1005508. 26348919 10.1371/journal.pgen.1005508 PMC4562644

[32] Liu G , Wang L , Pan J . Chlamydomonas WDR92 in association with R2TP‐like complex and multiple DNAAFs to regulate ciliary dynein preassembly. J Mol Cell Biol. 2019;11:770‐780. 30428028 10.1093/jmcb/mjy067 PMC6821370

[33] Huang B , Piperno G , Luck DJ . Paralyzed flagella mutants of *Chlamydomonas reinhardtii*. Defective for axonemal doublet microtubule arms. J Biol Chem. 1979;254:3091‐3099. 429335

[34] Horani A , Gupta DK , Xu J , et al. The effect of Dnaaf5 gene dosage on primary ciliary dyskinesia phenotypes. *JCI* . Insight. 2023;8:8. 10.1172/jci.insight.168836 PMC10393236 37104040

[35] Matsuo M , Shimada A , Koshida S , Saga Y , Takeda H . The establishment of rotational polarity in the airway and ependymal cilia: analysis with a novel cilium motility mutant mouse. Am J Physiol Lung Cell Mol Physiol. 2013;304:L736‐L745. 23525783 10.1152/ajplung.00425.2012

[36] Ha S , Lindsay AM , Timms AE , Beier DR . Mutations inDnaaf1andLrrc48Cause Hydrocephalus, Laterality Defects, and Sinusitis in Mice. G3 (Bethesda). 2016;6:2479‐2487. 27261005 10.1534/g3.116.030791 PMC4978901

[37] Kumar V , Umair Z , Kumar S , Goutam RS , Park S , Kim J . The regulatory roles of motile cilia in CSF circulation and hydrocephalus. Fluids Barriers CNS. 2021;18:31. 34233705 10.1186/s12987-021-00265-0 PMC8261947

[38] Ji W , Tang Z , Chen Y , et al. Ependymal cilia: physiology and role in hydrocephalus. Front Mol Neurosci. 2022;15:927479. 35903173 10.3389/fnmol.2022.927479 PMC9315228

[39] Lee L . Riding the wave of ependymal cilia: genetic susceptibility to hydrocephalus in primary ciliary dyskinesia. J Neurosci Res. 2013;91:1117‐1132. 23686703 10.1002/jnr.23238

[40] Hoque M , Chen D , Hess RA , Li FQ , Takemaru KI . CEP164 is essential for efferent duct multiciliogenesis and male fertility. Reproduction. 2021;162:129‐139. 34085951 10.1530/REP-21-0042 PMC8269963

