# Supplementary material for: A novel homozygous mutation in the DNAAF3 gene leads to severe asthenozoospermia and teratospermia
Source: J Cell Mol Med. 2024 Sep 17;28(18):e70092. doi: 10.1111/jcmm.70092 (PMC11408122; doi:10.1111/jcmm.70092)
Supplement: Supplementary file 1 — Table S1. [file JCMM-28-e70092-s001.docx]

**Supplementary Table 1. Body weight of homozygous mice and wild-type mice**

| Group | No. | Body weight, g | Mean ± SD, g^a^ |
| --- | --- | --- | --- |
| Homozygous mice | 1 | 5.86 | 6.49±0.93 |
|  | 2 | 7.55 |  |
|  | 3 | 6.05 |  |
| Wild-type mice | 1 | 15.91 | 15.50±0.37 |
|  | 2 | 15.38 |  |
|  | 3 | 15.21 |  |

^a^ The body weight of homozygous mice was significantly lower than that of wild-type mice (6.49±0.93 vs. 15.50±0.37 g, P=0.0015).

**Supplementary Table 2. Testicular volume of homozygous mice and wild-type mice**

| Group | No. | a, cm | b, cm | c, cm | Volume, cm^3 a^ | Volume, mm^3^ | Mean ± SD, mm^3 b^ |
| --- | --- | --- | --- | --- | --- | --- | --- |
| Homozygous mice | 1 | 0.168 | 0.107 | 0.107 | 0.008056853 | 8.056853122 | 8.90±0.74 |
|  | 2 | 0.148 | 0.122 | 0.122 | 0.009227201 | 9.227201106 |  |
|  | 3 | 0.132 | 0.1305 | 0.1305 | 0.009416371 | 9.41637106 |  |
| Wild-type mice | 1 | 0.254 | 0.147 | 0.147 | 0.022990954 | 22.99095416 | 27.28±4.03 |
|  | 2 | 0.226 | 0.1715 | 0.1715 | 0.027843594 | 27.84359431 |  |
|  | 3 | 0.2375 | 0.1765 | 0.1765 | 0.030991432 | 30.99143192 |  |

a The testicular volume of mice was calculated based on the ellipsoid volume formula: V= 4πabc/3, where a, b, and c represent half of the lengths of the three axes of the ellipsoid.

b The testicular volume of homozygous mice was significantly lower than that of wild-type mice (8.90±0.74 vs. 27.28±4.03 mm^3^, P<0.0001).
